# Proliferative verrucous leukoplakia is associated with aggressive periodontal pathogens: a comparative study with oral lichen planus and chronic periodontitis cases

**DOI:** 10.1007/s00784-025-06525-9

**Published:** 2025-09-23

**Authors:** Jan Liska, Veronika Liskova, Nikoleta Molnarova, Ondrej Topolcan, Petr Posta, Lukas Hauer

**Affiliations:** 1 Faculty of Medicine in Pilsen, Charles University, Alej Svobody 1655/76, 32300 Pilsen, Czechia; 2 Department of Stomatology, University Hospital Pilsen, Alej Svobody 80, 30460 Pilsen, Czechia; 3 Central Laboratory of Immuno-analysis, Faculty of Medicine in Pilsen, University Hospital, Charles University, Ed. Beneše 13, 30599 Pilsen, Czechia; 4 Biobank of Faculty of Medicine in Pilsen, Charles University and of University Hospital Pilsen, Ed. Beneše 13, Pilsen, 30599 Czechia

**Keywords:** Proliferative verrucous leukoplakia, Oral lichen planus, Periodontal pathogens, Desquamative gingivitis, DNA test

## Abstract

**Objectives:**

This study compares the proportional presence and the differences in means and medians of periodontal pathogens in cases of proliferative verrucous leukoplakia (PVL) versus patients diagnosed with oral lichen planus (OLP) with desquamative gingivitis (DG) and those with chronic periodontitis (CP).

**Materials and methods:**

The study evaluated the presence of periodontal pathogens in 38 PVL cases verified by clinicopathological criteria, an equal number of histologically verified DG cases, and another 38 cases of CP. All patients were treated at the Oral Medicine Department of the Dentistry Clinic, Pilsen Faculty Hospital, between 2012 and 2024. All cohorts experienced CP with a similar level of progression (moderate, with approximately 6-millimeter-deep pockets). Twelve different periodontal pathogens were identified using DNA tests (VariOr-Dento). The study focused primarily on the aggressive pathogens classified within the red and orange complexes. The status of periodontal resorption was diagnosed through orthopantomograms and clinical examinations of periodontal pockets.

**Results:**

The proportional dominance of red and orange complex periodontal pathogens in PVL was evident compared to gingival OLP and CP cases. PVL had the highest calculated risk for periodontal resorption and a greater mean number of aggressive bacteria per microliter.

**Conclusion:**

Our research underscores the significant association between aggressive red and orange complex periodontal pathogens and PVL. In our study, PVL exhibited a threefold higher number of these bacteria than controls with CP. These findings may highlight an important cofactor in the pathogenesis of PVL.

**Supplementary Information:**

The online version contains supplementary material available at 10.1007/s00784-025-06525-9.

## Introduction

**Fig. 1:**
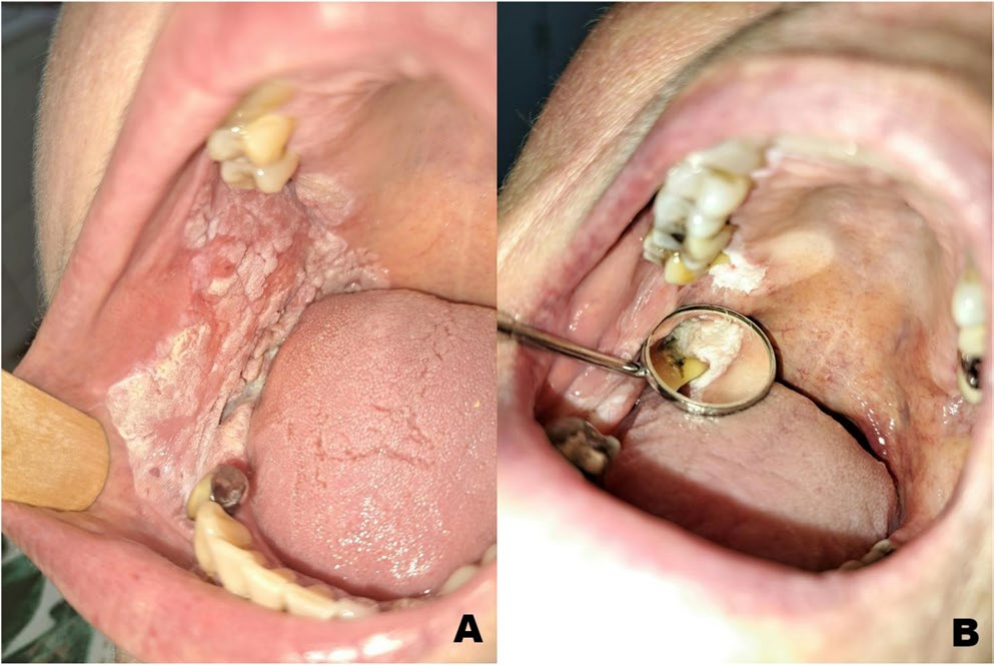
Demonstration of Proliferative verrucous leukoplakia (PVL) on the buccal mucosa (Female, 61) and alveolar ridge (Female, 72); a) PVL on the buccal mucosa: a rare oral mucosal finding with clinical significance; b) PVL on alveolar ridge: the most common PVL occurrence affecting both gingiva and alveolar mucosa. Both lesions progressed to carcinoma

Proliferative Verrucous Leukoplakia (PVL) is a rare and distinct form of leukoplakia, characterized by heterogeneous, wart-like white patches on the oral mucosa [1–4]. The condition was first described by Hansen in 1985 [5]. Clinically, PVL typically progresses from small, unifocal leukoplakia to multifocal lesions with continuous growth and a high tendency for malignant transformation [3]. Lesions most commonly appear on the alveolar mucosa, including both attached and free gingiva, as well as on the tongue; they are less frequently found on the floor of the mouth, palate, or buccal mucosa (Fig. 1) [1, 3]. Silverman described a subtype involving only the gingival surface, called gingival PVL [6]. The differential diagnosis of PVL includes oral lichen planus (OLP), traumatic hyperkeratosis, alveolar ridge keratosis, unifocal epithelial dysplasia, and chronic hyperplastic candidiasis [2, 3].

The mean age of patients with PVL is 66.8 years, with a strong predominance of women (ratio to men of 2.72:1) [1]. Histopathological examination demonstrates hyperkeratosis, hyperplasia, acanthosis, DNA aneuploidy, and lymphocytic infiltration adjacent to the basal membrane [1, 3]. Advanced lesions present with a verruciform surface. The dysplastic profile differs from dysplastic-free epithelial hyperplasia, ranging from mild dysplasia to severe dysplasia, followed by the appearance of frank oral carcinoma. The lesion is not reactive to friction, trauma, or topical agents [2].

The etiology of PVL is still unknown [1, 3]. Hansen associated this lesion with smoking, but subsequent studies have found it to occur in non-smokers as well [1, 3, 5]. Human papillomavirus has been suspected as an inducer of PVL but has not been frequently verified in biopsies [1]. Some studies report a higher incidence of *Candida* hyphae in biopsies, but this feature is likely due to secondary contamination [1].

Cases of PVL are verified through clinical–histopathological correlation using specific criteria. The diagnostic criteria for PVL, as outlined by Cerero-Lapiedra [1, 3, 5, 7] (Table 1), are divided into five major and four minor criteria. To establish a diagnosis of PVL, a lesion must meet either three major criteria (histopathological compatibility is mandatory) or two major criteria plus two minor ones [7].

PVL has a median time to malignant transformation (MT) into oral squamous cell carcinoma (OSCC) of six years [1]. Gingival PVL can transform into OSCC or verrucous carcinoma [1, 2, 6]. The overall malignant transformation rate exceeds 50%, with an annual MT rate of up to 10% [2, 8–12]. This behavior is significantly more aggressive than that of simple leukoplakia, which has an MT rate ranging from 0.13 to 17.5% [3].

PVL is classified as one of the oral potentially malignant disorders (OPMDs) recognized by the WHO [1, 6, 11, 13–24]. It has the highest proportional MT rate among all OPMDs, followed by erythroplakia [16, 20]. The most common sites for MT in PVL are the gingiva and the palate [19].

**Table 1:** Cerero-Lapiedra criteria for proliferative verrucous leukoplakia

Major criteria	Minor criteria
Leukoplakia lesions affecting more than two different oral sites, most frequently the gingiva, alveolar processes, and palate.	Oral leukoplakia lesions with a total affected area of at least 3 cm.
Presence of a verrucous area.	Female patient.
Lesions that spread or engross during the disease development.	Non-smoker patient.
Recurrence in a previously treated area.	Disease duration of more than 5 years.
Histopathological findings ranging from simple epithelial hyperkeratosis to oral squamous cell carcinoma (either in situ or invasive).	

Numerous therapeutic approaches have been attempted, but none have achieved lasting success [1, 9]. Surgical excision is often combined with laser ablation [1, 5]– [6]; however, scalpel excision is generally preferred, as it allows for more accurate histopathological assessment of the biopsy specimen [1]. Hansen also explored the use of radiotherapy and chemotherapy, though without consistent or sustained improvement [1, 5]. Extensive surgical resection is typically reserved for cases in which MT has occurred [1].

In aggressive cases, close monitoring—including frequent follow-ups and repeat biopsies—is essential due to the progressive nature of the lesions [1, 9]. Even patients without recurrence should be scheduled for biannual outpatient clinic checkups [1].

What remains largely outside the current diagnostic and therapeutic focus is the condition of the tissue adjacent to PVL lesions. Periodontal tissue lies in close proximity not only to the gingival surface but also to the margins of the tongue and buccal mucosa. While the complexity of the subgingival microbiome has been recognized for decades, only recently has the intricate relationship between aggressive periodontal pathogens and their interactions with both nearby and distant organs gained wider attention [25].

The coordinated development of aggressive pathogens such as *Tannerella forsythia* (*T. forsythia*), *Porphyromonas gingivalis* (*P. gingivalis*), and *Treponema denticola* (*T. denticola*) has been identified using DNA probe technologies [25]. These three bacteria constitute the so-called red complex, which is associated with the most severe clinical manifestations of periodontitis. Additional pathogenic synergy has been observed between *Fusobacterium nucleatum* (*F. nucleatum*) and *Parvimonas micra* (*P. micra*), along with *Prevotella intermedia* (*P. intermedia*), *Eubacterium nodatum* (*E. nodatum*), and *Campylobacter rectus* (*C. rectus*), which are members of the orange complex [25].

The detection of aggressive periodontal pathogens is essential for assessing the risk of periodontal tissue resorption and is critical for establishing optimal therapeutic strategies [26]. The pathogens of the red complex, along with *Filifactor alocis* (*F. alocis*), are key contributors to tissue destruction in chronic periodontitis, producing enzymes such as proteases and exotoxins. These bacterial products are directly responsible for the degradation of periodontal tissue and the induction of inflammation. The pathogenicity of these organisms increases proportionally with their concentration.

Bacteria are responsible not only for initiating inflammatory processes but also for promoting cancer development by downregulating apoptotic pathways and producing toxic components [27–31]. The link between chronic inflammation and carcinogenesis is well-established [27]. *P. gingivalis* is overrepresented in samples of gingival OSCC [27]. In cases of PVL, enrichment of potentially carcinogenic bacteria such as *T. forsythia* and *P. gingivalis*, along with marked dysbiosis of the microbiome, has been demonstrated [32, 33].

Bacterial activity, biofilm formation, and bacterial by-products sustain the pathological effects of chronic inflammation. Among the most important mediators are peptidases known as matrix metalloproteinases (MMPs) [34–37].

Antibiotics (ATBs), such as tetracycline or amoxicillin in combination with metronidazole, are used as systemic adjunctive treatments for periodontitis [26, 37]. MMP inhibitors, including tetracyclines, can counteract the effects of MMP activity in periodontal tissues [35]. The distinction in their use lies in the daily dosage and duration of the treatment regimen [38, 39]. For antimicrobial purposes, the typical dosage is 200 mg on the first day, followed by 100 mg daily; for MMP inhibition, a sub-antibacterial dose of 20 mg twice daily is used, usually for three to nine months [38–40].

This study aims to compare periodontal pathogens in rare PVL cases with those found in the more common condition of OLP associated with desquamative gingivitis and with age—and gender-matched patients diagnosed with CP. This comparison may offer insights into specific pathogenic cofactors involved in PVL, potentially contributing to improved therapeutic guidelines and prognosis for this disease. Our null hypothesis for this study is that periodontal pathogens have the same levels in periodontal pockets localized in different gingival pathologies.

## Materials and methods

Between 2012 and 2025, 76 cases of PVL were diagnosed and treated at the Dentistry Clinic in Pilsen with an average follow-up of 83 months (7 years).

### Inclusion criteria

Cases of PVL were confirmed both clinically and by biopsy, according to the Cerero-Lapiedra criteria. These criteria were categorized into major and minor and applied consistently across all PVL cases. To meet the inclusion threshold, patients needed either at least 3 out of 5 major criteria (average number of major criteria met: 4.02) or a combination of two major and two minor criteria (average number of minor criteria met: 2.5). No antibiotics were administered. No periodontal flap surgery was performed within the last three months. OLP cases included only those with desquamative gingivitis confirmed by both biopsy and direct immunofluorescence. CP cases were verified through clinical probing (identifying true periodontal pockets) and radiographic analysis of bone resorption. Only OLP and CP cases matched by age and gender with the PVL group were included. For the comparative study, only PVL, OLP and CP cases with periodontal damage assessed as moderate chronic periodontitis (1999 classification) or Stage III, Grade B periodontitis according to the 2017 classification were included. By moderate chronic periodontitis were taken cases with a loss of connective tissue and bone with true periodontal pockets between 4 and 6 millimeters. Stage III periodontitis involves a periodontal pocket equal or deeper than 6 millimeters, possible loss of four or fewer teeth due to periodontitis. A vertical bone loss equal or deeper than 3 millimeters. Interradicular furcation up to third class can occur (furcation probing can go through and through). Grade B periodontitis means moderate progression rate of disease with loss of attachment less than 2 millimeters in 5 years.

### Exclusion criteria

Cases mimicking PVL that did not meet the Cerero-Lapiedra criteria were excluded. Those with PVL accompanied by conditions other than moderate chronic periodontitis were excluded. OLP cases not confirmed histologically were excluded. Patients with acute necrotizing ulcerative gingivitis, or those currently presenting with periodontal abscesses were also excluded. Patients who had used antibiotics or undergone periodontal flap surgery within the last three months were excluded. The cases of PVL, OLP or CP with different than moderate chronic/Stage III/Grade B state of periodontitis were excluded. Cases of former aggressive periodontitis, actually known as Grade C periodontitis and often associated with the presence of the bacterium *Aggregatibacter actinomycetemcomitans (A. actinomycetemcomitans)*, were excluded if this bacterium was verified by DNA test. All cases of PVL and OLP in edentulous patients were excluded. Finally, verified OLP or periodontitis cases not matched by age and gender with the PVL group were excluded.

A total of 38 patients initially diagnosed with PVL were excluded from this comparative study due to lack of follow-up, recent antibiotic use, or periodontal status not compatible with inclusion criteria.

PVL and OLP cases were diagnosed via a routine biopsy. When fungal infection was suspected, additional staining methods, such as periodic acid–Shiff (PAS) or Grocott’s methenamine silver stain, were used. In OLP cases, direct immunofluorescence was employed to detect fibrinogen deposits along the basement membrane. Desquamative gingivitis (characteristic gingival manifestation of OLP) can be generalized or localized. It is a combination of hyperkeratosis, atrophy and erosions by lichenoid destruction of the gingival epithelium. DG can be differentiated from clinical changes in CP by the absence of edema and hyperemia.

The PVL cases formed Group 1 (G1). Groups 2 (G2) and 3 (G3) comprised an equal number of patients: G2 included individuals with OLP and desquamative gingivitis, and G3 comprised patients with CP. All study groups deliberately had a prevalence of female patients as a reflection of the higher incidence of PVL and OLP in women, with patients with CP alone being matched according to groups G1, G2.

All participants provided informed consent for inclusion in the study and subsequent treatment. The cohort characteristics are presented in Table 2.

**Table 2:** Characteristics of study cohort

Group	Female/Male	Mean age	Age range
1: PVL	23/15	64.1	40–85
2: OLP with DG	23/15	63.9	37–83
3: Periodontitis	23/15	63.2	38–79

PVL: proliferative verrucous leukoplakia, OLP: oral lichen planus, DG: desquamative gingivitis

The condition of the periodontium was clinically assessed using a WHO periodontal probe and visualized with an orthopantomography. Alveolar bone resorption was assessed based on the distance between the cement-enamel junction and the marginal bone greater than 1 mm and the absence of compact bone. Tooth mobility was regularly monitored. Periodontal pathogens were identified by their DNA, extracted from subgingival plaque samples collected with five sterile paper points in each case. After the procedure were the paper point put into the sterile Eppendorf test tubes to avoid contamination. Only periodontal pockets deeper than 5 millimeters were analyzed. The average depth of these pockets was 5–6 millimeters. To avoid bias was the probing performed by one experienced clinician by WHO probe in all present teeth.

The VariOr-Dento Plus system (Gen-Trend Co.) was used to differentiate 12 pathogens responsible for alveolar bone resorption by their relative proportions and to quantify their concentration in microliters. Bacterial DNA was detected using qPCR with a TaqMan probe. The sensitivity of the DNA probe was set to 10¹. The maximum proportional score for pathogen presence was 4, indicating a high risk of periodontal attachment loss, corresponding to a pathogen count greater than *n* = 10⁶. The score decreased to 1, representing a lower resorptive risk, with bacterial counts around *n* = 10³.

Data were processed using SigmaXL software and analyzed with the non-parametric Kruskal–Wallis method.

## Results

PVL lesions were most frequently located on the alveolar ridge, affecting the gingiva or alveolar mucosa in 34% of cases (*n* = 44/129), followed by lesions on the tongue and buccal mucosa, each occurring in over 20% of cases. Lesions on the floor of the mouth, labial mucosa, and palatal mucosa were rare, each accounting for less than 10%.

Nonsmokers comprised 46% of PVL patients (*n* = 35/76). The follow-up revealed that carcinoma developed in 38% of PVL patients (*n* = 29/76). The most common site for oral squamous cell carcinoma (OSCC) was the alveolar ridge, accounting for 45% of cases (*n* = 13/29).

The comparative study included 38 patients diagnosed with PVL after excluding 38 cases based on exclusion criteria—primarily due to the absence of pathogen testing. The female-to-male ratio in this study cohort was 1.53:1 (*n* = 23/15). The average age of PVL patients at the time of periodontal pathogen testing was 64.1 years (range 40–85). Given that the majority of patients were over 60 years of age and that approximately 40% of the study groups were male, the influence of female sex hormones on the development of pathological findings was not evaluated.

**Fig. 2:**
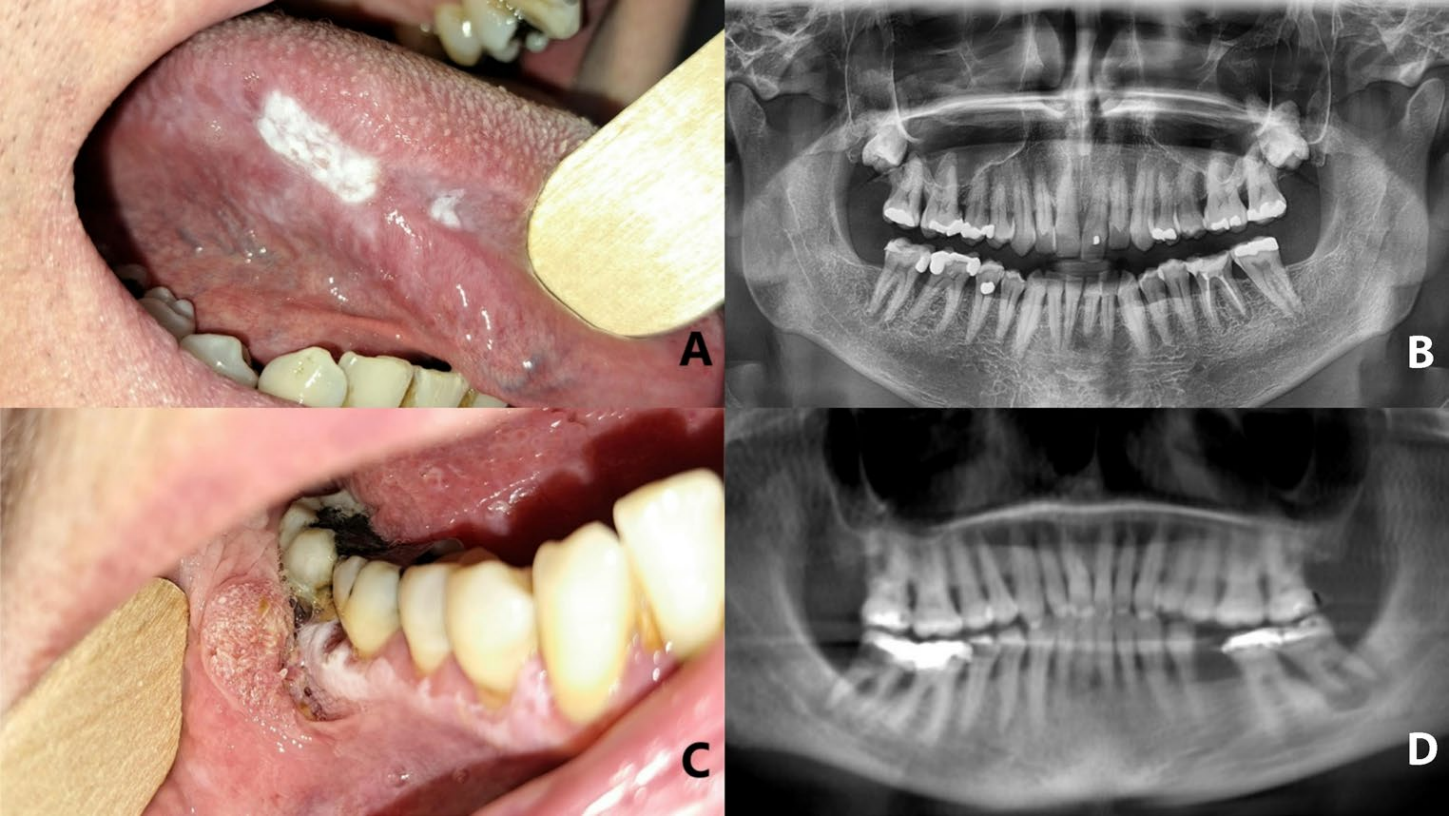
Correlation of clinical locations of proliferative verrucous leukoplakia (PVL) lesion with alveolar bone resorptions. On the margin of the tongue (Male, 50) and alveolar ridge (Female, 69); (a) PVL on the margin of the tongue and (b) resorption distal to tooth 46; (c) PVL on alveolar ridge and (d) resorptions in 47 and 37 locations

The addition of a comprehensive periodontal examination improved the observed correlation between PVL lesion locations and the distribution of periodontal resorption. This relationship is illustrated in Fig. 2.

The proportional values for each of the 12 examined bacteria and their counted number were compared. Mean values and medians from volumes were calculated. Table 3 presents a comparison of the percentage proportions of red and orange complex pathogens (the highest values are shown in bold).

**Table 3:** Percentage proportional comparison of red + orange complex periodontal pathogens

Disease x Pathogen	Pg	Tf	Td	Fa	Pm	Pi	Fn
PVL	**59.2**	**78.3**	**63.8**	**40.1**	**61.8**	**39.5**	**74.3**
OLP w/DG	55.3	64.5	44.7	22.4	59.9	26.3	66.4
Periodontitis	53.9	65.1	52.6	32.9	55.9	32.2	68.4

Pg: Porphyromonas gingivalis, Tf: Tannerella forsythia, Td: Treponema denticola, Fa: Filifactor alocis, Pm: Parvimonas micra, Pi: Prevotella intermedia, Fn: Fusobacterium nucleatum

## Discussion

Our study brings a new insight into the comprehensive problem of PVL pathogenesis. As far as we know, this is the first comparative study with this objective. The threefold increase of aggressive periodontal pathogens in comparison to CP significantly complicates the immune response in cases of PVL.

Proliferative verrucous leukoplakia (PVL) is a rare clinical form of oral potentially malignant disorder (OPMD) [13, 16, 19, 24]. It has been included in the WHO classification of OPMDs from the 3rd to the 5th edition [24]. The most recent (5th) edition, published in 2022, categorizes OPMDs to include PVL, leukoplakia, erythroplakia, oral epithelial dysplasia (OED), HPV-associated OED, and oral submucous fibrosis [24].

PVL lesions are typically idiopathic, often multifocal, and characterized by aggressive clinical behavior, a high recurrence rate, and a significant risk of malignant transformation—posing considerable challenges for therapeutic management [1, 3, 7, 24]. In the early stages, PVL may resemble flat leukoplakia; histologically, it can present with lichenoid infiltration, leading to potential misdiagnosis as OLP [24].

While PVL was historically associated with smoking, several studies have confirmed its occurrence in non-smokers [1, 3–5, 7, 11]. In our study, 46% of patients were non-smokers (*n* = 35/76). Alcohol consumption has been evaluated in the pathogenesis of PVL without significant findings [1, 11]; none of the patients in our cohort were categorized as regular alcohol consumers. Although a potential association with HPV has been discussed, no conclusive link has been established [1, 3, 9, 11]. Only 11% of our cases tested HPV-positive in our study. The presence of *Candida* hyphae is considered a secondary phenomenon [3]; in our cohort, 26% of biopsies were positive for *Candida albicans* staining. This may contribute to underdiagnosis, with PVL potentially mismanaged as chronic hyperplastic candidiasis in the absence of significant epithelial atypia.

PVL lesions are most commonly located on the alveolar crest, reported in up to 87% of cases [3, 14]. In our cohort, the distribution was broader, with alveolar involvement in 34% of cases, followed by the tongue and buccal mucosa. Clinical progression often begins at a single tooth’s gingiva and spreads across adjacent gum surfaces, peri-alveolar buccal, or lingual mucosa [4]. This behavior may reflect a pathogenetic process driven by aggressive periodontal bacteria. Thus, PVL management should involve not only regular clinical examinations and biopsies but also periodontal evaluation, as some lesions exhibit more aggressive behavior in the periodontium than on the epithelial surface. PVL is diagnosed using the Cerero-Lapiedra criteria, some of which are major and others minor [1, 3, 7, 14, 15]. Some authors have proposed simplified versions of these criteria for ease of use by less experienced clinicians [1, 4]. However, oversimplification risks omitting important indicators—such as female gender and non-smoking status—that are common in PVL patients and associated with high MT rates in the absence of classic risk factors. Applying the full Cerero-Lapiedra criteria is beneficial for early recognition and to avoid clinical underestimation [3]. Given PVL’s high progression rate to OSCC, management should be reserved for experienced clinicians.

Currently, no universally accepted treatment guideline ensures long-term disease control in PVL [1, 3, 9]. Approaches include surgery, laser ablation, photodynamic therapy, methisoprinol (Isoprinosine), and even chemoradiotherapy [5, 9]. While photodynamic therapy may successfully treat visible lesions, it does not prevent new lesion development [1]. In our study, conventional blade surgery remained the primary approach, supplemented by laser ablation and individualized antibacterial and antifungal therapy. For PVL progression affecting the gingiva, we opted to combine the excision of diseased soft tissue with the extraction of the involved tooth and thorough curettage of the socket to ensure the complete removal, including the crevicular epithelium [4]. Extensive resections were reserved for confirmed MT cases.

Recurrence rates for PVL in studies with over 30 participants range from 71.2 to 100% [3, 20]. Our recurrence rate was 65% (*n* = 49/76), which may be attributable to our aggressive surgical approach, particularly for gingival PVL, where gum and adjacent teeth excision was accompanied by alveolar bone revision. HPV-positive PVL cases showed fewer recurrences following a combination of surgery and methisoprinol (Isoprinosine) [9].

PVL has a malignant transformation rate exceeding 50% [2, 3, 8, 10–12, 17], significantly higher than that of conventional leukoplakia (0.13–17.5%) [3, 9, 15]. According to the 2020 WHO report, PVL exhibits the highest MT rate among all OPMDs [16]. Annual transformation rates reach up to 10%, compared to 1–5% in leukoplakia [3, 11]. Hansen et al. outlined ten stages of PVL progression toward OSCC; later authors condensed these into four by omitting intermediate stages [1, 5]. The “field cancerization” phenomenon is frequently observed in PVL [2]. The most common MT site in PVL is the gingiva or palate [19]; our data partially support this, with 45% of transformations (*n* = 13/29) occurring on the alveolar crest, though none were noted on the palate. Additional MT cofactors may include advanced patient age and frequent DNA aneuploidy in PVL biopsies [23]. Meta-analyses report an OSCC-related mortality rate of up to 21.29% in PVL cases [8, 19]. In our cohort, six patients (8%) died from PVL-associated OSCC (*n* = 6/76). The average follow-up duration in published studies is 7.8 years (range 3.7–11.6) [9]; our cohort had a mean follow-up of 7 years.

Growing evidence supports the role of periodontal pathogens in oncogenic processes at both local and distant sites [27–31]. Oral microbiome dysbiosis may influence carcinogenesis via chronic inflammation and the production of mutagenic metabolites [27, 29]. *F. nucleatum* is frequently associated with pancreatic and colorectal cancers [30], promoting inflammation, cellular proliferation and invasion, and immune evasion. *T. forsythia.* and *P. gingivalis* have been linked to esophageal cancer [27, 31]. *P. gingivalis* not only enhances the invasiveness of oral carcinoma cells but also contributes to chemotherapy resistance [27].

The pathogenicity of periodontal bacteria is not solely determined by bacterial load but also by their secreted products, such as MMPs. MMPs are zinc-dependent endopeptidases involved in the degradation of the basement membrane and extracellular matrix [34, 35] and are produced by both bacteria and host tissues. MMP-9, the largest of these enzymes, is elevated in neoplastic processes. It degrades type IV collagen and is thought to induce angiogenesis—an essential step in the development of OSCC from dysplastic lesions [34]. MMP-9 is elevated in saliva samples from OSCC patients and may serve as a biomarker to distinguish them from those with OPMD or healthy controls [34, 36]. It also represents a potential target for therapeutic intervention [34].

A lingering question remains: why do bacteria, present in most individuals’ oral cavities, contribute to carcinogenesis in only select cases? The most likely explanation involves host-microbiome interactions and pathogenetic cofactors such as smoking, immunosuppression, or viral co-infection [30]. Members of the red complex are rarely found without co-existing orange complex bacteria [25]. The aggressive periodontal biofilm—comprising *A. actinomycetemcomitans*, *P. gingivalis*, *T. forsythia*, *T. denticola*, *P. intermedia*, and *F. nucleatum*—necessitates combined treatment, including antibiotics, as these pathogens can evade standard scaling procedures by invading adjacent tissues [26]. Biofilm formation increases bacterial resistance up to 500-fold [26]. In CP, the volume of red complex pathogens correlates with the depth of periodontal pockets [25]. This factor was carefully considered in our study’s inclusion and exclusion criteria to minimize bias.

Our results suggest that regular periodontal examinations and assessments of worsening periodontal status in alveolar PVL cases could aid in the early detection of malignant transformation. Incorporating antibacterial therapy and agents that inhibit bacterial byproducts may enhance treatment outcomes and prognosis, given that current therapies are largely ineffective and the rate of MT remains alarmingly high—reaching up to 38%.

Significant limitations of this study include the rarity of PVL cases and the frequent need for aggressive surgical intervention, which complicates the evaluation of therapeutic outcomes and impedes the establishment of standardized treatment guidelines. Another limitation lies in finding a balance between avoiding bias and maintaining enough cases to achieve significant results.

## Conclusion

Our research highlights a significant association between aggressive red and orange complex periodontal pathogens and PVL. In our study, PVL cases exhibited a threefold higher presence of these bacteria than controls with CP. These findings suggest an important cofactor in PVL pathogenesis.

**Fig. 3:**
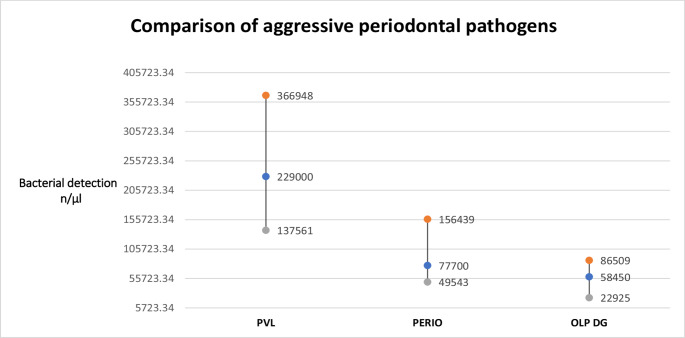
Graph showing proportional detection of red + orange complex periodontal pathogens. The maximum and minimum values for PVL cases exceeded those found in clinical cases of CP and chronic mucosal and gingival OLP lesions

**Fig. 4:**
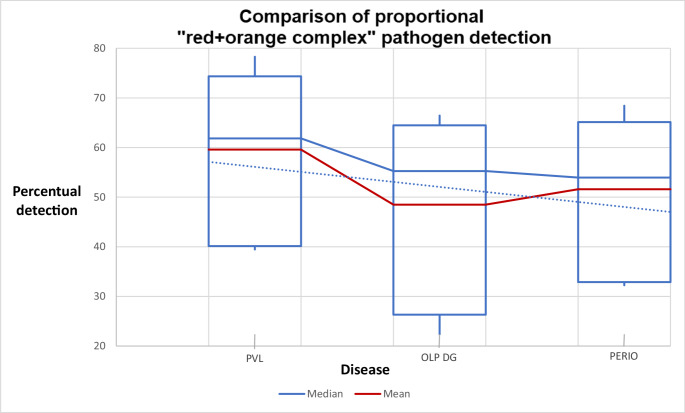
Graph comparing red + orange complex periodontal pathogens. A significant association with an aggressive oral microbiome in PVL is highlighted by an almost threefold increase in pathogen count compared to controls with chronic periodontitis

PVL cases showed dominance over the other two groups in terms of proportional ratios, mean and median counts of each individual pathogen from the red and orange complexes, and risk ratio for periodontal resorption. This difference was statistically significant, with *p* = 0.0009 for proportional detection (Kruskal-Wallis test), and confidence intervals as follows: PVL (3–3), Periodontitis (2–3), and OLP (2–2). For bacterial volume (in microliters), the statistical significance was *p* = 0.0003 (Kruskal-Wallis), with confidence intervals: PVL (CI: 138k–367k), Periodontitis (CI: 50k–156k), and OLP w/DG (CI: 23k–87k). Results of both comparisons are presented in Figs. 3 and 4.

The mean ratio of pathogen volume was 2.95:1:0.75 for PVL: Periodontitis: OLP, indicating an almost threefold higher presence of aggressive periodontal pathogens in PVL cases compared to chronic periodontitis. Our null hypothesis has been disproved.

The inclusion of routine periodontal examination improved the clinical management of alveolar PVL lesions. Early detection of MT has become more feasible, even in cases that initially appear clinically stable.

## Supplementary Information

Below is the link to the electronic supplementary material.

Supplementary Material 1

## Data Availability

The raw data for the dataset of the study are available in the supplementary file.
